# Hypertension Assessment Using Photoplethysmography: A Risk Stratification Approach

**DOI:** 10.3390/jcm8010012

**Published:** 2018-12-21

**Authors:** Yongbo Liang, Zhencheng Chen, Rabab Ward, Mohamed Elgendi

**Affiliations:** 1 School of Electronic Engineering and Automation, Guilin University of Electronic Technology, Guilin 541004, China; liangyongbo001@gmail.com; 2 School of Electrical and Computer Engineering, University of British Columbia, Vancouver, BC V6T 1Z4, Canada; rababw@ece.ubc.ca; 3 Faculty of Medicine, University of British Columbia, Vancouver, BC V1Y 1T3, Canada; 4 BC Children’s and Women’s Hospital, Vancouver, BC V6H 3N1, Canada

**Keywords:** Hypertension, systolic blood pressure, photoplethysmograph, feature selection, risk classification

## Abstract

Hypertension is a common chronic cardiovascular disease (CVD). Early screening and diagnosis of hypertension plays a major role in its prevention and in the control of CVDs. Our study discusses the early screening of hypertension while using the morphological features of photoplethysmography (PPG). Numerous morphological features of PPG and its derivative waves were defined and extracted. Six types of feature selection methods were chosen to screen and evaluate these PPG morphological features. The optimal features were comprehensively analyzed in relation to the physiological processes of the cardiovascular circulatory system. Particularly, the intrinsic relation and physiological significance between the formation process of systolic blood pressure (SBP) and PPG morphology features were analyzed in depth. A variety of linear and nonlinear classification models were established for the comparison trials. The F1 scores for the normotension versus prehypertension, normotension and prehypertension versus hypertension, and normotension versus hypertension trials were 72.97%, 81.82%, and 92.31%, respectively. In summary, this study established a PPG characteristic analysis model and established the intrinsic relationship between SBP and PPG characteristics. Finally, the risk stratification of hypertension at different stages was examined and compared based on the optimal feature subset.

## 1. Introduction

In recent years, instances of cardiovascular diseases (CVDs) have been increasing worldwide due to a lack of awareness and a low rate of diagnosis and treatment. Meanwhile, the mortality rate of CVDs has overtaken that of cancer, making CVDs the leading cause of death in humans and, accordingly, a major public health problem [1]. The unintentional and unpredictable occurrence of acute CVDs [2], such as coronary artery disease, stroke, heart failure, and myocardial infarction, can have irreversible consequences and may produce many sequelae; whereas, chronic CVDs, such as hypertension, hyperlipidemia, and atherosclerosis, cause slow deterioration, thereby leading to a greater potential for delayed treatment as well as the need for lifelong medication. Many CVDs require long-term health care and protection, which consumes large amounts of medical and health resources; consequently, such CVDs represent a global health challenge.

As an important physiological index of the cardiovascular system, blood pressure (BP) plays a major role in predicting the occurrence of acute and chronic CVDs [3,4]. Therefore, the early screening and evaluation of patients with hypertension or prehypertension is of great practical significance [5,6]. Hypertension [7] in adults over 18 years of age is defined as systolic or diastolic BP that is consistently higher than an acceptable normal value (currently 139 mmHg systolic, 89 mmHg diastolic). Different high BP levels are accompanied by different risks; therefore, hypertension has been subdivided into stage 1 hypertension and stage 2 hypertension. The criterion for stage 1 hypertension is systolic blood pressure (SBP) that is in the range of 140 to 159 mmHg or diastolic blood pressure (DBP) in the range of 90 to 99 mmHg. The criterion for stage 2 hypertension is SBP exceeding 160 mmHg or DBP exceeding 100 mmHg. The measures used to determine normotension are based on the evolutionary trend of human BP and have been subdivided into normotension and prehypertension. The criterion for normotension is SBP ranging between 90 to 119 mmHg and DBP ranging between 60 to 79 mmHg concurrently. The criterion for prehypertension is SBP ranging from 120 to 139 mmHg or DBP ranging from 80 to 89 mmHg. It is well known that BP fluctuates with age. For those with hypertension, without proper control and management, they will most likely need treatment. Therefore, the management and control of hypertension is critical to reducing its severity; additionally, numerous non-drug methods, such as lifestyle changes and exercise, can significantly improve BP, returning it to normal levels.

BP is currently measured with an electronic sphygmomanometer [8]. This measurement method has been widely recognized and popularized over the last century of development and it has played a major role in the control of CVDs. However, both the Korotkoff sound [9] method and the oscillometric method [10] require cuffs and pressure to the forearm when BP is measured, which means that the measurement can easily be affected by operation and use conditions, such as the operation of the cuff, sitting posture, and exercise. In addition, it also has a definite white coat phenomenon (BP is higher when it is taken in a medical setting than it is when taken at home) for some patients [3]. Therefore, new cuff-less hypertension screening and BP detection technologies are under development [11]. 

Photoplethysmography (PPG) provides abundant physiological information [12] about the operation of the cardiovascular circulation system at a low cost and with convenient signal acquisition; therefore, it is of great interest to researchers. A series of in-depth investigations [13,14,15,16,17] have been carried out to non-invasively assess BP, blood sugar, stiffness, and fatigue levels using PPG signals. The common PPG acquisition methods can be divided into transmission and reflection methods [18]. A PPG signal is extracted by an LED transmitter section, which generates a red or infrared light to illuminate the skin of the fingertip, earlobe, or forehead, and a photosensitive diode, which measures the time-varying light absorption of the tissue and it can reflect changes in blood volume. Therefore, PPG is the external manifestation and aggregated expression of various physiological processes in the cardiovascular circulation system. PPG is a high fusion signal that covers the activity of the heart’s systolic and diastolic periods [19] and the hemodynamic [20], hemorheological [21], and network information of the peripheral microcirculation system [22].

In recent years, more features of the PPG signal have been proposed and validated to predict BP or evaluate hypertension [23], such as pulse transit time [16], pulse wave velocity [23], augmentation index [5], stiffness index, PPG intensity ratio [24], and time-domain characteristics [25]. Meanwhile, arterial wave propagation theory [16] and PPG morphological theory have also been researched: The former is based on ECGs or other physiological signals and the PPG signal, while the latter is based on PPG morphological features. However, more research focused on the healthy subjects and the limited number of hypertensive subjects. Therefore, BP readings and PPG signals were collected for this study from relatively large number of healthy and hypertensive subjects [26,27].

To clarify hypertension risk stratification using only one non-invasive biosignal, the authors have conducted extensive research in this area [28,29,30,31]. We have found that two approaches can be implemented: a feature-based method [28,29] or a non-feature-based method [31]. For the sake of providing a simple model suitable for wearable devices, we have only focused on the feature-based method. Therefore, our research design for this study can be outlined, as follows: (1) the physiological relevance of the PPG signal was comprehensively analyzed using the feature-based method (depending on the PPG waveform morphology), (2) the PPG features were selected and ranked using machine learning algorithms, (3) multiple linear and nonlinear models were investigated for hypertension risk stratification, and (4) a portable health device and matching app were developed based on the internet of things, wearable technologies, and Android platforms for ubiquitous hypertension screening and tracking.

## 2. Materials and Methods

### 2.1. Data Acquisition

**Figure 1:**
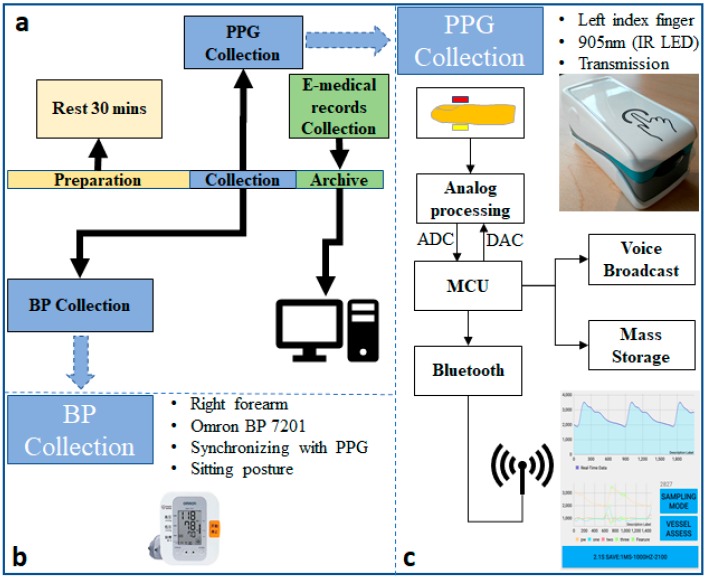
The block diagram of data collection. (**a**) Data collection protocol; (**b**) Blood pressure (BP) collection; (**c**) photoplethysmography (PPG) collection and the customized hardware platform.

In this study, a portable hardware platform was designed, consisting of a PPG probe and matching mobile phone app; the data were transmitted via Bluetooth. For the PPG probe, an MSP430FG4618 MCU was embedded on the board to configure the ADC and DAC and to fetch the data for transmission to the matching app via Bluetooth. The mobile phone app has several functional modules, namely the photoelectric control module, the waveform real-time display module, the sampling precision configuration module, and the data storage module. For the purposes of this study, the PPG signal acquisition was configured as an infrared (905-nm wavelength) transmission mode with a sampling rate of 1 kHz and a 12-bit ADC, and the hardware filter design was 0.5–12 Hz bandpass. Figure 1 shows a diagram of the data collection process and the customized hardware platform.

Data collection was performed on 219 subjects recruited from Guilin People’s Hospital [26] and was conducted according to the ethics rules and regulations of both the hospital and the Guilin University of Electronic Technology in China after approval by the ethics committee. Participants who suffered from diseases, such as neurological disorders were excluded, as were those suffering from diabetes (38 subjects), cerebral infarction (20 subjects), cerebrovascular disease (37 subjects), or those who were taking hypotensive drugs during the experimental period. In the experiment, participants first waited in a rest area for more than 30 min before entering the data collection room. After entering the room, they were seated in a chair with a backrest in the most comfortable posture and instructed to place their arms flat on a blank desktop. The operators simultaneously used a customized probe to collect the PPG signal from the participants’ left index fingers and an Omron 7201 electronic sphygmomanometer to determine BP from their right forearms. The PPG and BP acquisition process was completed within 3 min. Three 2.1-s-long PPG waveform data records were saved in the matching app for each participant. Additionally, physiological information from the participants, including gender, age, height, and weight, was also collected through the app, and disease records were obtained through the hospital’s electronic medical records. More details about the background and introduction of the project can be found in [26]. The dataset has been fully uploaded to the network and users can download it via this link [26].

Before the experiment, all of the participants were fully informed about the experimental content and their informed consent was obtained. In addition, before the data were archived, we removed personal identifiers. Ultimately, we generated a BP database that includes data from 124 subjects (age: 55 ± 16; Height: 163 ± 8 cm; Weight: 62 ± 15 kg; 65 males and 59 females). The risk stratification approached was implemented by dividing the dataset according to the hypertension classification criteria: hypertensive group (SBP ≥ 140 or DBP ≥ 90), prehypertensive group (SBP 120–140 mmHg or DBP 80–90 mmHg), and normotensive group (SBP < 120 and DBP < 80). The hypertensive group included 35 subjects; the prehypertensive group included 41 subjects; and, the normotensive group included 48 subjects.

### 2.2. PPG Signal Pre-Processing

The raw PPG signal was first pre-processed by a PPG signal quality evaluation [32], a bandpass filter, and a mathematical derivative [13]. The PPG signal quality evaluation was conducted via the skewness method for the three waveform records of each subject. Elgendi [32] compared eight different signal quality indices: P_SQI_, skewness (S_SQI_), kurtosis (K_SQI_), entropy (E_SQI_), signal-to-noise ratio (N_SQI_), zero-crossing (Z_SQI_), matching of multiple systolic wave detection algorithms (M_SQI_), and relative power (R_SQI_). For lengths of PPG waveforms between 2 s and 30 s, the S_SQI_ method demonstrated better performance than the other methods (P_SQI_, K_SQI_, E_SQI_, N_SQI_, M_SQI_, Z_SQI_, and R_SQI_). Using this method, the classifications of excellent or unfit waveforms were best when 3 s was used as the window of the PPG waveform segment. In this study, the S_SQI_ method was used to evaluate the records, and only the segment with the highest S_SQI_ value for each subject, which included the PPG feature analysis and the BP classification model, was selected. For all the selected waveform records, a fourth order 0.5 Hz–10 Hz bandpass Chebyshev II filter was used to filter noise and improve the PPG signal quality while, at the same time, the PPG features were kept undistorted [27]. Subsequently, two times the mathematical derivative of the PPG signal was implemented to acquire the velocity plethysmogram (VPG), the acceleration plethysmogram (APG), and the third derivative waveform (third derivative), all of which were used to define and extract the characteristics of the PPG signal.

### 2.3. Feature Extraction

A PPG signal contains information on the heart’s systolic and diastolic activity, hemodynamics, and hemorheology. Therefore, in a PPG signal and its derivatives, many potential physiological characteristics [12,33] can be defined or quantified. Some of these characteristics have been found and confirmed by a large number of researchers; for example, the characteristics of the systolic and diastolic periods, crest time, augmentation index, and large artery stiffness index [33]. There are other characteristics that have potential physiological feasibility, but these have not yet been confirmed in clinical data and have therefore not yet been valued. 

**Figure 2:**
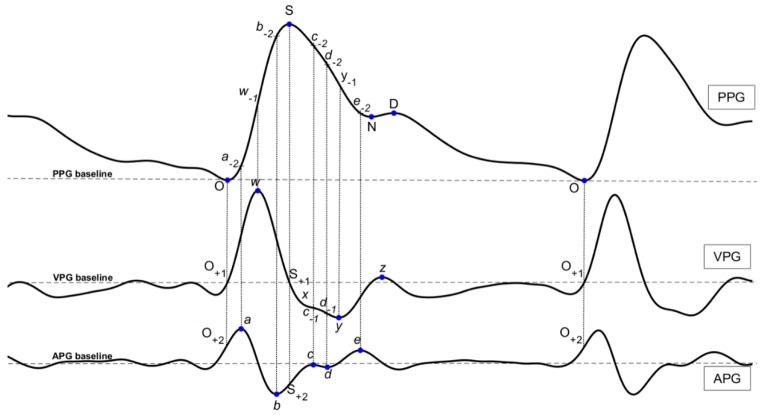
The definition of the characteristics in PPG and its derivatives.

In this study, a comprehensive investigation of the characteristics of the PPG signal and its derivatives was conducted according to the recommendation in [30]. For consistency and clarity, we have suggested the following waveform names: “Onset (O)”, “Systolic Peak (S)”, “Notch (N)”, and “Diastolic Peak (D)” in the PPG waveform; “*w* wave (*w*)”, “*x* wave (*x*)”, “*y* wave (*y*)”, and “*z* wave (*z*)” in the VPG waveform; and “*a* wave (*a*)”, “*b* wave (*b*)”, “*c* wave (*c*)”, “*d* wave (*d*)”, and “*e* wave (*e*)” in the APG waveform. The *b*, *c*, and *d* waves appearing in the PPG and VPG waveforms were named “*a*_−2_”, “*b*_−2_”, “*c*_−2_”, “*d*_−2_”, and “*e*_−2*”*,_ and “*c*_−1_” and “*d*_−1_”, respectively. All of the wave names are marked in Figure 2. The characteristics’ features based on their wave names were defined and include several types, such as time span (23 features), PPG amplitude (14 features), features of VPG and APG (10 features), waveform area (4 features), power area (15 features), ratio (43 features), and slope (16 features). A total of 125 features are defined and described in this paper.

#### 2.3.1. Time Span 

The time span features are expressed as their letters with a dash over each. For example, the $\bar{SD}$ feature represents the time span from the systolic peak S to the diastolic peak D. A total of 23 time-span features were defined.

#### 2.3.2. Features of PPG Amplitude

The S, N, D, *w*_−1_, *a*_−2_**,** *b*_−2_, and *c*_−2_ features were defined in the PPG waveform. These features represent the amplitude of the corresponding waveform from the PPG baseline. For example, the S amplitude feature represents the S height from the PPG baseline to the systolic peak S, and the N amplitude feature represents the N height from the PPG baseline to the diastolic notch N. Other features that are based on these features were also defined, such as N/S, *w*_−1_/S, *b*_−2_/S, and *c*_−2_/S. A total of 14 PPG amplitude features were defined.

#### 2.3.3. Features of VPG and APG

The *a*, *b*, *c*, *d*, and *e* features were defined in the APG waveform (the automatic detection of these waves is discussed here: [34,35,36,37]), and the *w*, *x*, *y*, and *z* features were defined in the VPG waveform. These features represent the amplitude of the corresponding waveform from the APG baseline and the VPG baseline. Other features based on these features were also defined, such as *b*/*a*, *c*/*a*, *d*/*a*, *e*/*a*, (*b*−*c*−*d*−*e*)/*a*, and (*b*−*c*−*d*)/*a*. A total of 20 VPG and APG features were defined.

#### 2.3.4. Waveform Area

The waveform AC component area features are expressed as their letters with a polyline over each. For example, the $\hat{\mathrm{OS}}$ feature represents the AC component area under the curve from the onset point O to the systolic peak S. A total of four waveform area features were defined.

#### 2.3.5. Power Area

The waveform AC component power area features are expressed as their letters with a brace over each. For example, the $\overset{︷}{\mathrm{OS}}$ feature represents the quadratic sum of the curve point from the onset point O to the systolic peak S. A total of 15 waveform area features were defined.

#### 2.3.6. Ratio

The ratio features are expressed directly as their ratio formulae. For example, the $\bar{OS}/\bar{OO}$ feature represents the ratio of the time span feature $\bar{OS}$ and the time span feature $\bar{OO}$, and the $\hat{OS}/\hat{OO}$ feature represents the ratio of the curve area feature $\hat{OS}$ and the curve area feature $\hat{OO}$. A total of 43 ratio features were defined.

#### 2.3.7. Slope

The slope features are expressed as their letters with a tilde over each. For example, the $\tilde{\mathrm{OS}}$ feature represents the slope from the onset point O to the systolic peak S. A total of 16 slope features were defined.

### 2.4. Feature Selection Methods

Before feature selection processing, z-score normalization was conducted for all extracted features. The mean and variance of the features were 0 and 1, respectively. The common feature selection methods included filtering, wrapping, and embedding; these methods were used to analyze the importance of the features for classification or prediction purposes from different angles. In this study, we used filtering feature selection methods [38,39], including the Spearman correlation [40], ReliefF [41], information gain (Info Gain) [39], chi-square, mRMR [42], and the Gini index. Relief is a feature selection algorithm that was first proposed for binary classification by Kira and Rendell in 1992 [41]. This algorithm chooses instances randomly and changes the weights of the relevant feature based on the nearest neighbor. Kononenko [41] proposed the ReliefF method, which is based on Relief, to solve multi-class classification. Generally speaking, ReliefF is one of the most successful strategies in feature selection. Information gain [39] is frequently used in feature selection in the field of machine learning. The information gain method regards each feature as an isolated variable and calculates its information gain; then, it evaluates the importance and relevance of each feature with a class label. The chi-square test is used to examine the dependency of the target variable (i.e., SBP) and each PPG feature; in this study, if the SBP variable was independent of each feature, then we could discard that feature variable. Conversely, if the SBP variable was dependent, then the feature variable was very important.

The minimum redundancy maximum relevance (mRMR) method [42] is a scheme that is used in feature selection to select the features most strongly correlated with a classification variable and remove the redundant features. This scheme, combined with selection features that are mutually distinct yet maintain a high correlation, comprise the selection scheme of mRMR. This combined scheme has been found to be more powerful than maximum relevance selection alone. The Gini index is a statistical measure of dispersion that is based on another statistical phenomenon, called the Lorentz curve, and it is commonly used to quantify wealth distributions, although its applications are limitless.

### 2.5. Classification Model

Based on the analysis of the PPG features, this study established four common linear and nonlinear classification learner models to classify the hypertension population: linear discriminant analysis (LDA), cubic SVM, weighted KNN, and logistic regression (LR).

**Figure 3:**
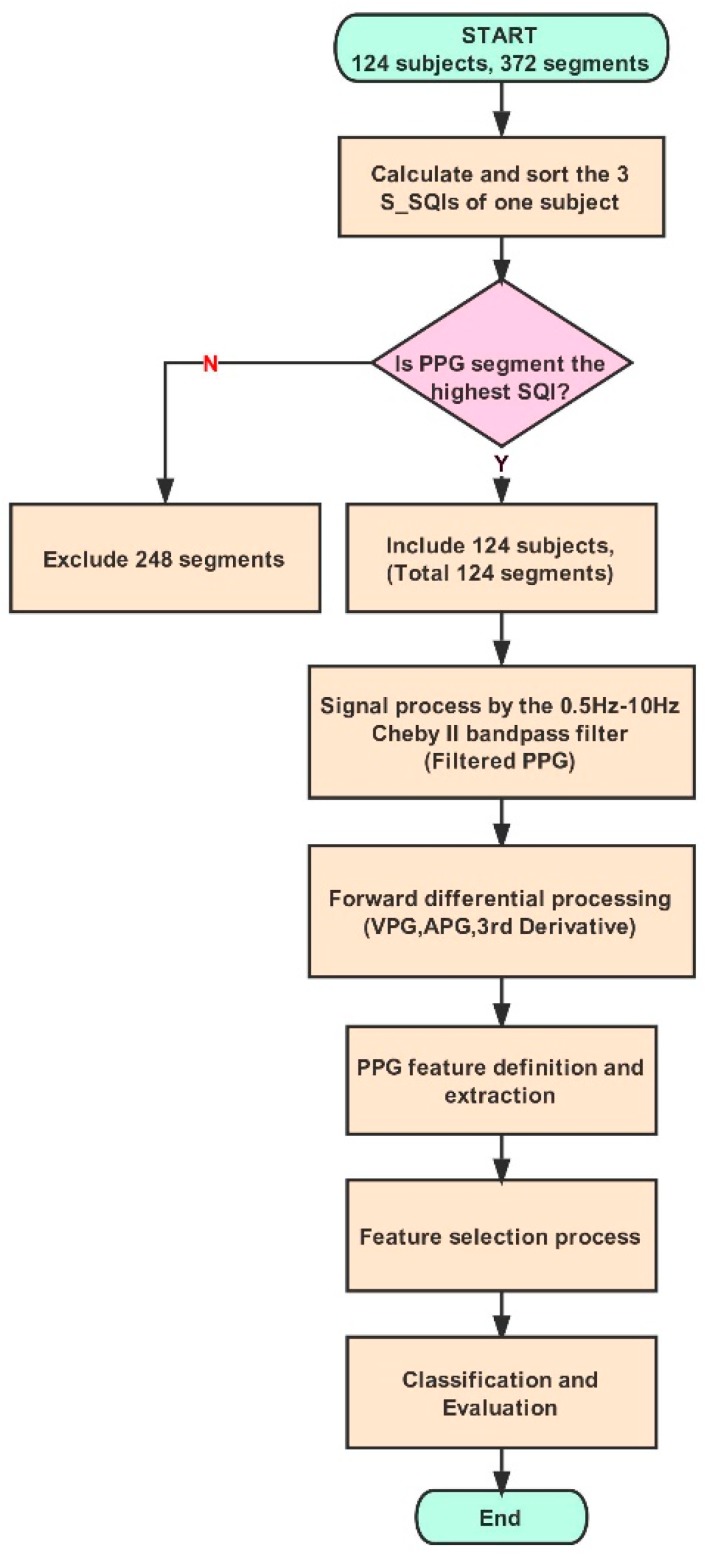
The flowchart of PPG signal process and hypertension classification.

An optimal feature subset composed of the 10 features that were selected by the feature selection method was trained and tested, and the 10-fold cross-validation was configured to evaluate the performance of the classification model. Figure 3 shows the workflow of the signal process and classification model. The data processing and modeling estimations were carried out using MATLAB software (Version R2017a, MathWorks, Inc., Natick, MA, USA).

## 3. Results

### 3.1. Correlation Coefficient between the SBP and Features

**Figure 4:**
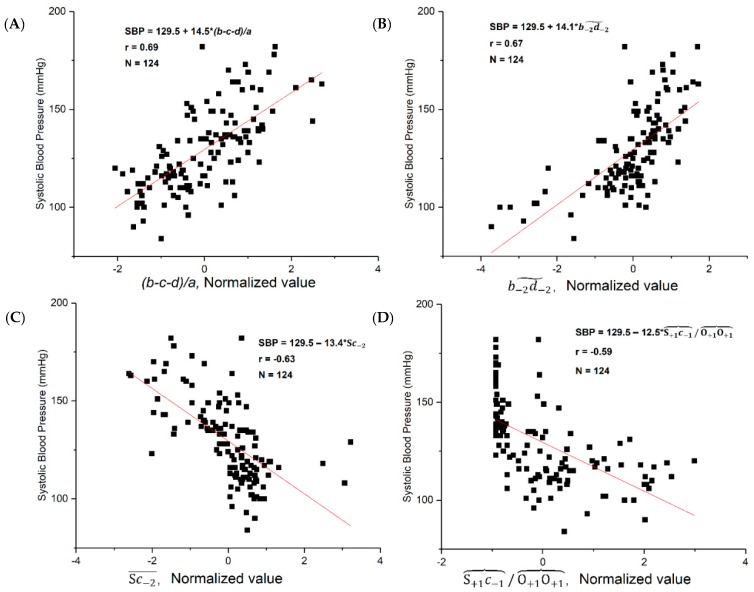
The regression analysis of the four features with the strongest correlation with systolic blood pressure (SBP). (**A**) (*b*-*c*-*d*)/*a* (**B**) $\tilde{b_{-2}d_{-2}}$ (**C**) $\tilde{Sc_{-2}}$ (**D**) OS.

Many studies [19,43,44,45,46,47] have shown that there is a linear relationship between some parameters of the PPG signal and SBP. First, the correlations between SBP and the selected features were examined. We found that several correlation coefficients were greater than 0.5 between the features and SBP. The *p* values of these features showed a significant difference (*p* < 0.01). This demonstrated that some PPG features have a significant correlation with SBP and they can therefore be used as biomarkers for hypertension. Table 1 shows a correlation coefficient list of top 10 PPG features. Figure 4 shows the regression analysis of the four PPG features with the highest correlation with SBP. 

We tested 125 features, some were examined by other researchers [33], such as $\bar{\mathrm{OS}}$, which is also called crest time (CT). Other, new features are defined and examined in this paper. From the features with strong correlations, we found that the most informative features lied within the interval from the *b* wave to the *d* wave. As is well-known, BP is a major physiological parameter of the human body. The features that are defined in this paper reflect the phenomenon of BP, which is important for the indirect measurement of BP and the risk stratification of the hypertensive population.

### 3.2. Top 10 Features Ranking

From the above correlation analysis, we found that numerous features have a significant correlation with SBP. Beyond the correlation study, we also evaluated the contribution of PPG features to hypertension classification via several feature selection methods. Table 2 shows the 10 selected features that were processed with six feature selection methods in hypertension classification. These selection methods are based on different mathematical models. Therefore, the selected features are slightly different.

### 3.3. Model Result

The optimal feature subset, which includes the top 10 features of each method selection, was produced via the various feature selection methods described above. The 10-fold cross-validation was used. For the evaluation index of risk stratification performance [48], the sensitivity (SE), positive predictive (PP), and F1-score (F1) were used to conduct the comparison and performance evaluation of the models. 

Three risk stratification levels were performed: the normotensive group (48 subjects) versus the prehypertensive group (41 subjects), the normotensive group (48 subjects) versus the hypertensive group (35 subjects), and the normotensive and prehypertensive group (89 subjects) versus the hypertensive group (35 subjects). Table 3 shows the risk stratification performance results in the risk stratification process using the top 10 features. 

As mentioned above, six feature selection methods were adopted to evaluate the important features. Based on the ranked features, multiple classifications were conducted using the top 10 features. As we have seen in this paper, KNN (*K* = 10) showed better performance in classifying the different BP categories. From Table 3, it can also be seen that KNN achieved better classification performance results than the other classifiers. 

It is worth mentioning that we investigated the top 20 features as well, with the aim of improving overall accuracy. However, the classification performance results based on the top 20 features were not better than those that were based on the top 10 features. Accordingly, our conclusion was that more features did not improve the performance of the risk stratification. Therefore, in this paper, the top 10 features were analyzed and applied toward classifying the BP categories.

## 4. Discussion

We know that human blood vessels and microcirculation systems undergo changes with increases to BP. These changes are especially obvious for patients with severe hypertension. PPG signals contain abundant information that can reflect the change process. Therefore, high-quality signal acquisition of PPG signals is very important for accurately assessing the intrinsic relationship between BP and PPG signals. 

The SBP of human arteries is the maximum value that is formed in the middle of the systolic period under the action of the fusion of blood propulsion and reflection from the heart. Therefore, the propulsion and reflection fusion process of arterial blood during the systolic period plays an important role in the formation of SBP. The accurate analysis of this physiological process via the PPG signal is of great significance for detecting or evaluating the systolic pressure level. 

The PPG signal reflects physical changes of blood volume in blood vessels during cardiac activity and blood transmission, and it is the direct representation of the blood volume state [49,50]. The VPG signal reflects the extent of blood volume change in the peripheral blood vessels and it is the manifestation of blood change [13]. The APG signal reflects the accelerated change of blood volume, and this phenomenon largely reflects the systolic and diastolic ability of the heart. In addition, the moment of propulsion and reflection is very important for analyzing the fusion process of the propulsion wave and the reflection wave, which can be more prominent in the APG signal. Therefore, PPG, VPG, APG, and the third derivative can reflect cardiac activity in the vascular circulatory system from different dimensions, and therefore the systematic and coherent analysis of waveform signals is very important in understanding the physiological process more clearly. 

From the feature selection analysis, we also found that features with a strong correlation to systolic pressure and a greater contribution weight with BP labels were concentrated in the interval between the systolic and diastolic periods, specifically in the interval between the *b* wave and the *c* wave. The *b* wave represents the smallest moment of acceleration for the main propulsion wave and it occurs when the main propulsion wave reaches its peak (S). In contrast, the *c* wave is the largest moment of acceleration for the reflected wave. This interval shows significant differences in the PPG waveform due to different BP levels. Therefore, the study and analysis of the physiological changes of blood vessels in the *b* wave and *c* wave periods can help to clarify the formation of the systolic pressure peak more accurately. Most importantly, the precise comprehension of this process is very useful for improving the accuracy of systolic pressure detection. In addition, signal sampling accuracy needs special attention. Generally, the interval between the *b* wave and *c* wave is about 60–130 ms (median: 90 ms) and it accounts for 7–14% (median: 11%) of a heartbeat cycle. Therefore, low signal sampling accuracy loses a substantial amount of important information. To avoid any under-sampling issues, such as ignoring critical PPG features in the data collection phase, we used a high sampling frequency rate of 1 kHz with a high AD conversion rate of 12 bits.

In this study, some features reflected the details of the fusion process of the main propulsion wave and the reflected wave, such as $\bar{Sc_{-2}}$, ***c*_−2_**/S, $\tilde{Sc_{-2}}$, $\overset{︷}{S_{+1}c_{-1}}/\overset{︷}{O_{+1}O_{+1}}$, and $\tilde{b_{-2}d_{-2}}$. In Table 1, the top 10 features include two time span features, two slope features, three power areas, and three amplitude ratio features. Seven features’ correlation coefficients of the top 10 features were greater than 0.6. More importantly, the features are mainly related with the *b*, S, *c*, and *d* waves. As is well-known, the S wave and *c* wave exhibit different morphological changes under different physiological statuses, especially different BP levels [13,30]. Therefore, the selected features in this study are good expressions of the conditions of the main propulsion wave and reflected wave. This result confirms our findings on the MIMIC database, which indicate that the *bd* area is associated with hypertension [28]. 

We know that the moment of the fusion of the reflection wave and the main propulsion wave has a decisive effect on the actual SBP. The early fusion of the reflection wave and the main propulsion wave leads to an increase in human BP, while delayed fusion generates adverse effects on the maintenance of the ideal level (90–120 mmHg/60–80 mmHg) of BP. The features in this study have clearer physiological explanations than *a*, *b*, *c*, *d*, *e*, and (*b*−*c*−*d*−*e*)/*a*. The features of *a*, *b*, *c*, *d*, and *e*, and their combination, reflect the ability of blood propulsion in the blood vessels, which can in turn reflect the physiological process of BP formation. 

However, the features in this study directly reflect the formation of BP under the influence of multiple factors, and they are thus closer to the physiological significance of BP; therefore, they serve as the theoretical and physiological basis for the realization of non-cuff continuous ambulatory BP measurement. The characteristic expressions of physiological phenomena in the cardiovascular circulation process are of great significance to the development of non-invasive BP detection techniques and the non-invasive evaluation of cardiovascular aging and other physiological phenomena. Note that the approach achieves the six-step biomedical signal analysis framework for tackling non-communicable diseases, such as hypertension [51].

In Table 2, most of the useful features were selected by different feature selection methods, which are based on different theories. Here, ReliefF combined with the KNN classifier achieved better risk stratification performance results than the other models. ReliefF is an excellent feature weighting method for the classification problem. Less relevant features are removed, while more relevant features are retained, and all of the features are evaluated one by one, with the selected features representing those that are most useful for classification. In addition, as was shown earlier, the experimental results of the normotensive group versus the hypertensive group were better than those of the normotensive group versus the prehypertensive group and the normotensive and prehypertensive groups versus the hypertensive group. 

We know that in the treatment and management of hypertension [4,52], doctors generally do not provide drug or medical treatment but instead advise patients to adjust their diets and lifestyles to restore normal BP levels. In some patients, diet and lifestyle changes are enough, and medical therapy may not be necessary, especially in prehypertensive or stage 1 hypertensive patients [7]. However, medication is needed to sufficiently reduce BP for most stage 1 and almost all stage 2 hypertension cases [7]. Stage 2 hypertension cases also require longer-term treatment and drug control than stage 1 hypertension cases [7]. 

Hypertension in different stages can lead to varying degrees of cardiovascular circulation change; some changes are mild or reversible (unstable changes, such as prehypertension or stage 1 hypertension), while others are qualitative (chronic changes, such as stage 2 hypertension and severe hypertension, for which it is difficult to restore the normal BP level). Therefore, the cardiovascular circulatory system will exhibit different reflections for different BP levels [53]. 

One limitation of our study was that we examined the correlation of PPG features with only SBP. Finding the optimal features for detecting DBP and mean arterial pressure is our next step. Moreover, we must still investigate the classification between prehypertension and hypertension.

For chronic changes in the cardiovascular system, the risk stratification can show higher performance; in contrast, for reversible, unstable changes, which are prone to fluctuations in BP, the risk stratification shows relatively low performance. This result also illustrates the significance of long-term, dynamic BP monitoring. Yet, there are some limits to this approach. In this study, the sampling rate of the device was configured as 1 kHz. A high sampling rate and greater sampling precision consume more power, and therefore the sampling frequency must be reduced to save power so that the device can collect data for a longer period of time. As is known, the power consumption of wearable or portable devices is high. Therefore, low power consumption and a more efficient algorithm design must be achieved. In addition, the dataset was collected from an experimental room in which noise and other types of interference were avoided as much as possible. Thus, future work should be conducted in real-world conditions.

## 5. Conclusions

In this paper, we discussed hypertension risk stratification that was based on the morphological features of PPG signals and machine learning technology. By analyzing the physiological significance of the PPG features, the quantitative expression of SBP formation was realized, and the intrinsic relation between SBP formation and PPG features was established. By using six feature selection methods, 10 PPG features were found to be associated with systolic pressure. We observed the effects of different BP levels on physical changes in the cardiovascular circulatory system in the hypertension classification experiments with the optimal feature subsets, and these changes were more directly reflected in the PPG signal and its derivative signals. The risk stratification model showed higher performance in detecting severe hypertension than non-severe hypertension (such as prehypertension). In the PPG signal, a certain variability and instability were also observed. Therefore, higher accuracy in long-term, dynamic monitoring and evaluation for detection or screening is more realistic. The method that is described in this paper can hopefully achieve ambulatory BP measurement based only on the PPG signal. In future studies, we will develop and examine the proposed method for long-term monitoring of BP.

## Figures and Tables

**Table 1:** A correlation coefficient list of top 10 features with systolic blood pressure (SBP).

Index	1	2	3	4	5	6	7	8	9	10
Feature	(*b*-*c*-*d*)/*a*	$\tilde{b_{-2}d_{-2}}$	$\tilde{Sc_{-2}}$	$\bar{OS}$	*c*_−2_/S	$\overset{︷}{S_{+1}c_{-1}}$	$\overset{︷}{S_{+1}c_{-1}}/\overset{︷}{O_{+1}O_{+1}}$	*b*_−2_/S	$\bar{Sc_{-2}}$	$\overset{︷}{wS_{+1}}/\overset{︷}{O_{+1}O_{+1}}$
Correlation coefficient	0.6903	0.6721	0.6181	0.6164	0.5332	−0.4722	−0.5928	−0.6353	−0.6391	−0.6482
*p*-value	0.0001	0.0009	0.0013	0.0017	0.0425	0.0443	0.0058	0.0004	0.00003	0.0079

**Table 2:** The 10 selected features processed with six feature selection methods in hypertension risk stratification, which are ranked in descending order.

Feature Rank	Spearman	ReliefF	Info Gain	Chi2	mRMR	Gini
1	$\tilde{b_{-2}d_{-2}}$	$\overset{︷}{S_{+1}c_{-1}}/\overset{︷}{O_{+1}O_{+1}}$	$\bar{Sc_{-2}}$	$\bar{Sc_{-2}}$	(*b*−*c*−*d*)/*a*	*c*_−1_/*w*
2	(*b*−*c*−*d*)/*a*	$\bar{Sc_{-2}}$	(*b*−*c*−*d*)/*a*	*c*_−1_/*w*	(*b*−*c*−*d*−*e*)/*a*	$\overset{︷}{S_{+1}c_{-1}}/\overset{︷}{O_{+1}O_{+1}}$
3	$\tilde{Sc_{-2}}$	$\overset{︷}{S_{+1}d_{-1}}/\overset{︷}{O_{+1}O_{+1}}$	*c*_−2_/S	$\overset{︷}{S_{+1}c_{-1}}/\overset{︷}{O_{+1}O_{+1}}$	*c*_−1_/*w*	$\bar{Sc_{-2}}$
4	$\bar{OS}$	*c*_−2_/S	(*b*−*c*−*d*−*e*)/*a*	$\tilde{b_{-2}d_{-2}}$	$\overset{︷}{Sc-}/\overset{︷}{OO}$	$\tilde{b_{-2}d_{-2}}$
5	*c*_−2_/S	*c*_−1_/*w*	*c*_−1_/*w*	(*b*−*c*−*d*)/*a*	$\bar{Sc_{-2}}$	$\bar{Sd_{-2}}$
6	$\overset{︷}{S_{+1}c_{-1}}$	(*b*−*c*−*d*)/*a*	$\overset{︷}{S_{+1}c_{-1}}/\overset{︷}{O_{+1}O_{+1}}$	(*b*−*c*−*d*−*e*)/*a*	$\tilde{b_{-2}d_{-2}}$	*c*_−1_
7	$\overset{︷}{S_{+1}c_{-1}}/\overset{︷}{O_{+1}O_{+1}}$	*d*	$\tilde{b_{-2}d_{-2}}$	$\bar{Sd_{-2}}$	$\bar{Sd_{-2}}$	$\tilde{Sc_{-2}}$
8	*b*_−2_/S	$\tilde{b_{-2}d_{-2}}$	$\bar{Sd_{-2}}$	*c*_−2_/S	*c*_−2_/S	$\overset{︷}{S_{+1}c_{-1}}$
9	$\bar{Sc_{-2}}$	$\bar{OS}$	$\bar{OS}$	*c*_−1_	*c*_−1_	$\hat{Oc_{-2}}/\hat{OO}$
10	$\overset{︷}{wS_{+1}}/\overset{︷}{O_{+1}O_{+1}}$	$\tilde{Sc_{-2}}$	*c*_−1_	$\tilde{Sc_{-2}}$	$\tilde{Sc_{-2}}$	(*b*−*c*−*d*)/*a*

**Table 3:** Risk stratification performance based on top 10 features. Three classifications are examined: Normotension (48 subjects) vs. Prehypertension (41 subjects), Normotension (48 subjects) vs. Hypertension (35 subjects), and Normotension + Prehypertension (89 subjects) vs. Hypertension (35 subjects). The classifier with the highest F1 score is highlighted in yellow. LDA stands for Linear Discriminant Analysis; SVM stands for Support Vector Machines, KNN stands k-nearest neighbors, and LR stands for Logistic Regression.

		LDA			LR			Cubic SVM			Weight KNN		
	Index	PP	SE	F1	PP	SE	F1	PP	SE	F1	PP	SE	F1
**Normal (*n* = 48)** **vs.** **Prehyp. (*n* = 41)**	**Spearman**	75.76%	60.98%	67.57%	70.27%	63.41%	66.67%	67.57%	60.98%	64.10%	75.00%	58.54%	65.75%
	**ReliefF**	71.79%	68.29%	70.00%	74.36%	70.73%	72.50%	65.12%	68.29%	66.67%	81.82%	65.85%	72.97%
	**Info Gain**	78.13%	60.98%	68.49%	73.53%	60.98%	66.67%	60.00%	51.22%	55.26%	80.65%	60.98%	69.44%
	**Chi2**	70.27%	63.41%	66.67%	72.73%	58.54%	64.86%	55.00%	53.66%	54.32%	75.00%	58.54%	65.75%
	**mRMR**	79.41%	65.85%	72.00%	76.47%	63.41%	69.33%	55.56%	60.98%	58.14%	71.43%	60.98%	65.79%
	**Gini**	70.59%	58.54%	64.00%	67.57%	60.98%	64.10%	58.54%	58.54%	58.54%	73.53%	60.98%	66.67%
**Normal (*n* = 48)** **vs.** **Hyp. (*n* = 35)**	**Spearman**	93.33%	80.00%	86.15%	91.18%	88.57%	89.86%	88.57%	88.57%	88.57%	90.32%	80.00%	84.85%
	**ReliefF**	93.55%	82.86%	87.88%	87.50%	80.00%	83.58%	96.77%	85.71%	90.91%	93.75%	85.71%	89.55%
	**Info Gain**	93.55%	82.86%	87.88%	82.86%	82.86%	82.86%	100.00%	82.86%	90.63%	93.55%	82.86%	87.88%
	**Chi2**	93.10%	77.14%	84.38%	87.88%	82.86%	85.29%	100.00%	77.14%	87.10%	96.67%	82.86%	89.23%
	**mRMR**	87.10%	77.14%	81.82%	78.38%	82.86%	80.56%	96.77%	85.71%	90.91%	100.00%	85.71%	92.31%
	**Gini**	93.33%	80.00%	86.15%	87.88%	82.86%	85.29%	100.00%	80.00%	88.89%	93.55%	82.86%	87.88%
**Normal + Prehyp. (*n* = 89)** **vs.** **Hyp. (*n* = 35)**	**Spearman**	59.26%	45.71%	51.61%	62.86%	62.86%	62.86%	65.52%	54.29%	59.38%	86.36%	54.29%	66.67%
	**ReliefF**	68.97%	57.14%	62.50%	60.00%	60.00%	60.00%	87.10%	77.14%	81.82%	87.50%	60.00%	71.19%
	**Info Gain**	69.57%	45.71%	55.17%	60.61%	57.14%	58.82%	58.33%	40.00%	47.46%	76.19%	45.71%	57.14%
	**Chi2**	64.00%	45.71%	53.33%	54.55%	51.43%	52.94%	76.00%	54.29%	63.33%	88.89%	45.71%	60.38%
	**mRMR**	73.08%	54.29%	62.30%	67.86%	54.29%	60.32%	52.50%	60.00%	56.00%	76.00%	54.29%	63.33%
	**Gini**	66.67%	51.43%	58.06%	59.38%	54.29%	56.72%	79.17%	54.29%	64.41%	84.00%	60.00%	70.00%
